# Novel Epidemic Metrics to Communicate Outbreak Risk at the Municipality Level: Dengue and Zika in the Dominican Republic

**DOI:** 10.3390/v14010162

**Published:** 2022-01-17

**Authors:** Rhys Kingston, Isobel Routledge, Samir Bhatt, Leigh R Bowman

**Affiliations:** Department of Infectious Disease Epidemiology, Imperial College London, St. Mary’s Campus, Norfolk Place, London W2 1PG, UK; rhys.g.kingston@gmail.com (R.K.); Isobel.Routledge@ucsf.edu (I.R.); bhattsamir@gmail.com (S.B.)

**Keywords:** dengue, Zika, arbovirus, modelling, reproduction number, epidemic, outbreak, Dominican Republic, early warning system, EWARS

## Abstract

Arboviruses remain a significant cause of morbidity, mortality and economic cost across the global human population. Epidemics of arboviral disease, such as Zika and dengue, also cause significant disruption to health services at local and national levels. This study examined 2014–2016 Zika and dengue epidemic data at the sub-national level to characterise transmission across the Dominican Republic. For each municipality, spatio-temporal mapping was used to characterise disease burden, while data were age and sex standardised to quantify burden distributions among the population. In separate analyses, time-ordered data were combined with the underlying disease migration interval distribution to produce a network of likely transmission chain events, displayed using transmission chain likelihood matrices. Finally, municipal-specific reproduction numbers (R_m_) were established using a Wallinga–Teunis matrix. Dengue and Zika epidemics peaked during weeks 39–52 of 2015 and weeks 14–27 of 2016, respectively. At the provincial level, dengue attack rates were high in Hermanas Mirabal and San José de Ocoa (58.1 and 49.2 cases per 10,000 population, respectively), compared with the Zika burden, which was highest in Independencia and San José de Ocoa (21.2 and 13.4 cases per 10,000 population, respectively). Across municipalities, high disease burden was observed in Cotuí (622 dengue cases per 10,000 population) and Jimani (32 Zika cases per 10,000 population). Municipal infector–infectee transmission likelihood matrices identified seven 0% likelihood transmission events throughout the dengue epidemic and two 0% likelihood transmission events during the Zika epidemic. Municipality reproduction numbers (R_m_) were consistently higher, and persisted for a greater duration, during the Zika epidemic (R_m_ = 1.0) than during the dengue epidemic (R_m_ < 1.0). This research highlights the importance of disease surveillance in land border municipalities as an early warning for infectious disease transmission. It also demonstrates that a high number of importation events are required to sustain transmission in endemic settings, and vice versa for newly emerged diseases. The inception of a novel epidemiological metric, R_m_, reports transmission risk using standardised spatial units, and can be used to identify high transmission risk municipalities to better focus public health interventions for dengue, Zika and other infectious diseases.

## 1. Introduction

Arboviruses are an informal name for a group of viruses transmitted by arthropods such as ticks, mosquitoes and sand-flies [1]—members of which include Rift Valley Fever, Chikungunya and West Nile Virus [2]. Arboviruses are commonly zoonotic and are the cause of increasing human disease burden worldwide. In recent years, the arboviruses Zika and dengue have afflicted millions via endemic and epidemic transmission, in part due to relatively few, effective means of control [3]. Indeed, current estimates suggest that the total annual burden of dengue infections is 390 million, with 96 million manifesting clinically [4]. Those at risk number 3.97 billion across 128 countries worldwide [5]. In the case of Zika, estimates of the global burden are not yet available; however, by the end of 2018, the Pan American Health Organisation (PAHO) had reported 19,020 suspected cases of Zika, with 1379 laboratory-confirmed cases in Brazil alone [6].

Dengue and Zika are principally transmitted via *Aedes* mosquitoes. When a female *Aedes* mosquito bites an infected human, the mosquito ingests a blood meal containing the virus, at which point the virus enters the mosquito midgut, proliferates and finally spreads to the salivary glands. Once the mosquito bites another person, the cycle is complete [7]. However, vertical transmission can also occur, and while this is rare for dengue [8] such transmission is more common with Zika; indeed, in a prospective cohort, 26% of maternal cases resulted in vertical transmission to the unborn foetus [9]. Importantly, sexual transmission between humans is also a significant driver of Zika epidemiology [10].

The basic reproduction number (R_0_) describes the average number of secondary infections produced by a single infectious individual in a totally susceptible population [11]. Epidemics involving novel pathogens are best described using R_0_, due to the absence of existing population immunity [12]. By contrast, R_eff_, the effective reproduction number (also known as the net reproduction number (R_n_)), is most appropriate in endemic settings [11] when part of the population is already immune [12]. In the absence of field data, mathematical modelling is used to average the expected number of new infections over all possibly infected individuals. This idea can be represented by a matrix where the reproduction number is recognised as the dominant eigenvalue of an operator, which is linear for every pair of functions, and can be calculated through modelling whilst considering other factors such as age stratification [13].

The simplest form of epidemiological modelling used is mechanistic, which deploys compartments with interconnected per capita rates to describe the movement of individuals in the population between diseased states [14]. This field has since been further expanded to include network analysis. Wallinga and Teunis applied this approach [15] to estimate both the serial interval distribution [16] and R_eff_ of the severe acute respiratory syndrome (SARS) epidemic. In similar research, Routledge et al., 2018, also used a network-based analysis to predict malaria elimination time scales [17]. Together, these studies further developed mathematical modelling used to calculate individual reproduction numbers [18] while building on the established Reed–Frost model of epidemic transmission [19,20]. While these approaches are powerful, they are reliant on granular data to infer geospatial disease spread at fine scales, yet these data are not always available.

Accordingly, this research sought to further analyse data in Bowman et al., 2018 [21], by describing the geospatial transmission of dengue and Zika using network reconstruction and the R_0_ at the regional level.

## 2. Study Aims

This research sought to describe the spatio-temporal relationship of dengue and Zika outbreaks between 2014 and 2016 at the sub-national level, by extending work by Bowman et al., 2018, which performed analysis at the national level [21]. The project had two broad aims: (1) to explore regions of high burden across the Dominican Republic; and (2) to develop modelling research on reproduction numbers and network reconstruction by constructing novel epidemic metrics that characterised the transmission contribution of larger geographical units.

## 3. Country Context: Dominican Republic

**Figure 1:**
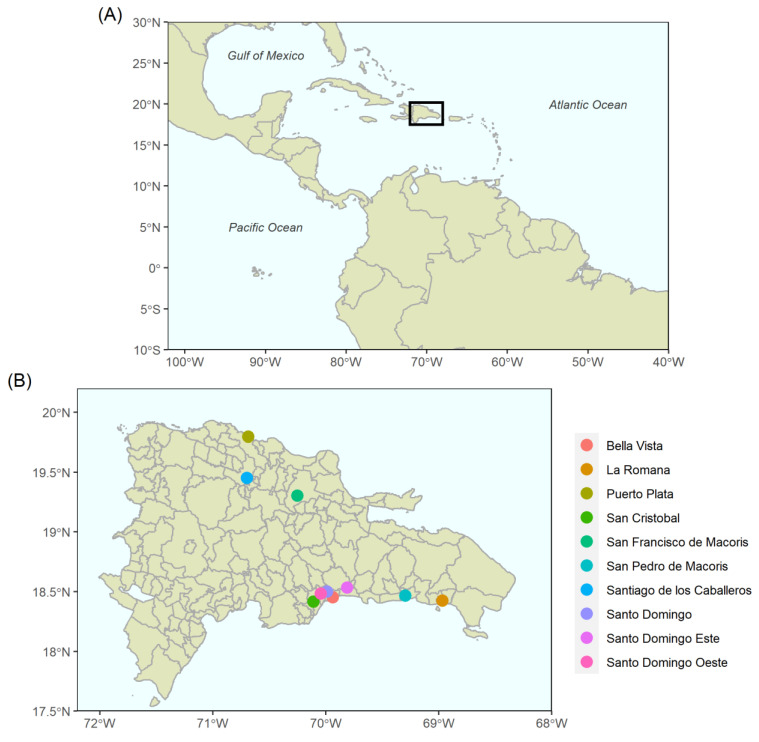

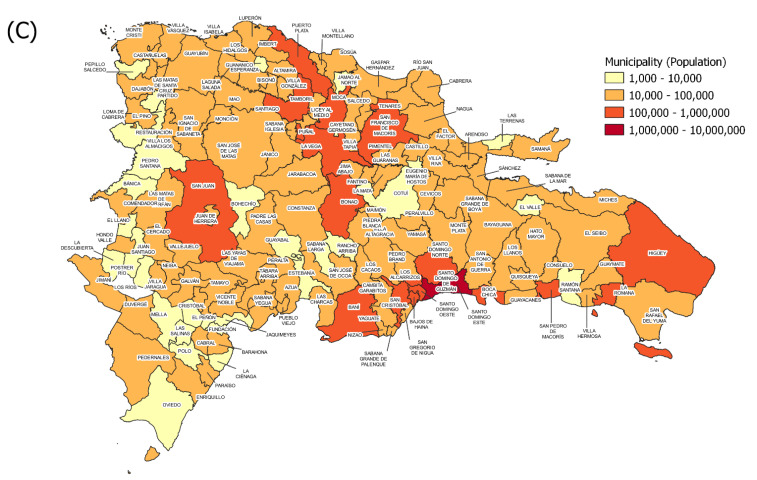
Mapping the Central and South American geographic area. (**A**) The location of the Dominican Republic on a continental scale. (**B**) Administrative municipal boundaries map of the Dominican Republic and ten most populous cities (not in order) [24]. (**C**) Administrative municipal boundaries map with 2016 population data, continuous colour scale from yellow (lowest) to red (highest). The shapefiles used for realisation of the Americas taken from the ‘ggplot2’ package and the Dominican Republic with administrative boundaries as they were in 2010, obtained from the Humanitarian Data Exchange [25,26].

The Dominican Republic is a country in the Greater Antilles region of the Caribbean [22], sharing an island with Haiti, as shown in Figure 1. According to demographic data released by the World Bank in 2016, the total population was 10.40 million over an area of 48.4 km^2^ [23].

The Dominican Republic is endemic for dengue, and recent research showed that 98% of the Santo Domingo population was seropositive [27]. In 2014–2016, a dengue and subsequent Zika outbreak occurred, during which large-scale control measures were deployed across the country. A range of clinical and epidemiological data were collected, providing an opportunity to study the progression of the outbreaks at the population level.

Bowman et al., 2018, used national surveillance data to generate descriptive statistics about the epidemics and found that 75% of dengue infections were in individuals less than 20 compared with a greater mean age of infection for Zika, largely as a function of population susceptibility [21]. Attack rates were calculated for each outcome variable and municipality—but it is not clear what effect over-reporting had, due to increased awareness from community campaigns [21].

## 4. Methods

### 4.1. Datasets

Surveillance data capturing cases of dengue, severe dengue and Zika were extracted from the Dominican Republic healthcare database, Sistema Nacional de Vigilencia Epidemiologica, for the years 2014 to 2016. Variables in the dataset included suspected/probable/confirmed cases, according to the 2009 WHO definition guidelines [28,29]; date of symptom onset; epidemiological week of onset; and date of notification; this in addition to other epidemiological variables of interest, such as age and sex. To capture aggregate dengue disease states, the following outcome variable labels were used: dengue (uncomplicated dengue); severe dengue (complicated dengue); and total dengue (complicated and uncomplicated dengue combined). Suspected incident dengue and Zika cases were used to form all outcome variables. Data were de-identified at the source, underwent quality control and cleaned as described in Bowman et al., 2016 [30], and Bowman et al., 2018 [21]. Population census data, stratified by age and sex for the years 2015–2017, were provided by the Oficina Nacional de Estadística. These data were used to standardise the attack rate calculations and to categorise the data into five-year age bins. All coding and analyses were performed in RStudio, version 3.6.0. [31], and all figures produced using the ‘ggplot2’ package [26].

### 4.2. Mapping

National surveillance data and population census data were used to plot the spatial distribution of suspected cases for all outcome variables per 10,000 population by municipality across the Dominican Republic. Maps were generated using shape files [25] with administrative boundaries from 2010. Maps plotted show the attack rate per 10,000 population (Equation (1)).

(1) $$Attack\;Rate=\frac{Case\;Count\;\left(in\;population\;of\;interest\right)}{Population\;of\;interest}\times 10000$$

### 4.3. Statistical Analyses

Age- and sex-standardised attack rates by province were calculated using a previously defined method [32]. The provincial populations were standardised to the population characteristics of the Dominican Republic using data provided by the Oficina Nacional de Estadística, with 95% confidence intervals (Equation (2)).

(2) $$Standardised\;Attack\;Rate\;\pm 1.96\times \frac{Standardised\;Attack\;Rate}{\#\;of\;events}$$

### 4.4. Disease Migration Interval Distribution

The disease migration interval is a novel parameter defined in this paper as the time between the onset of symptoms of the first case of each municipality. To calculate the distribution of potential intervals, a matrix of potential migration intervals was calculated by determining the non-negative differences between the initial symptom onsets within each municipality. The resultant distribution of the intervals then informed the probability density of infector, *i*, transmitting infection to the infectee, *j*. This interval reflects a higher-order version of the serial interval, which specifies the interval between symptom onset of the infector and infectee individual pairs. The benefit of using the serial interval in estimates is the ability to account for other important distributions of time in the transmission cycle, including the time from symptom onset to infectiousness, intrinsic incubation period, extrinsic incubation period and mosquito transmission rate [17]. The probability density of the disease migration interval was fitted to an exponential distribution after visualisation of the data strongly indicated an exponential trend. Fitting the distribution was achieved using maximum likelihood estimation with the exponential maximum likelihood estimator, seen below, where $\hat{\lambda_{n}}$ is the maximum likelihood estimator, n is the number of independent observations, x is a variable from an independent and identically distributed sample and $\overset{n}{\underset{j-1}{\sum }}x_{j}$ is the sum of all observations. The resultant simulated distribution was used to calculate the Wallinga–Teunis matrix (Equation (3)).

(3) $$\hat{\lambda_{n}}=\frac{n}{\sum_{j-1}^{n}x_{j}}$$

### 4.5. Determining the Transmission Likelihood and Network

The cases with the earliest symptom onset of dengue or Zika recorded within each municipality were identified, resulting in one case representing the earliest infection event for each municipality. These were ordered by date of symptom onset for each municipality, with no indication of the transmission chain present. Combination of this time-ordered data with a simulation of the underlying disease migration interval distribution produced a network of the most likely transmission chain events. This was achieved by analysing a network of all potential pairwise infector–infectee municipality pairs and their transmission likelihoods to isolate the most likely chain of transmission events across municipalities. The network of potential infector–infectee municipal pairs and their transmission likelihoods make up the Wallinga–Teunis matrix [15], made with the ‘IDSpatialStats’ package [33]. The matrix itself represents likelihood-based estimation of who-infected-whom using the observed dates of initial symptom onset of each municipality. Each square provides the relative likelihood, *p_ij_*, that the infector municipality, *i*, has infected an infectee municipality, *j*, given the time difference in symptom onsets of each municipality, *t_i_ − t_j_*. This time difference is captured by the disease migration interval distribution. As such, the relative likelihood that an infectee municipality, *i*, has been infected by an infector municipality, *j*, is the likelihood of this pair, normalised by the likelihood that the infector municipality, *i*, is infected by any other municipality, *k*. This analysis is based around the theory that infection events between the potential pairs follow an independent cascade model [34], where the upper triangular likelihood of the matrix equates total the realistic pairwise transmission likelihoods of the infector–infectee municipal pairs [15].

### 4.6. Estimating Time-Varying Municipal-Specific Reproduction Numbers

Municipal-specific reproduction numbers (R_m_) were established using the produced Wallinga–Teunis matrix wherein each column represents an infector municipality and each row represents an infectee municipality. To calculate the R_m_ for an infector municipality, *j*, we sum over all infectee municipalities, *i*, weighted by the relative likelihood that the infectee municipality, *i*, has been infected by the infector municipality, *j*. At a municipal level, this reflects the average number of secondary infectee municipalities arising from a primary infector municipality. This can be interpreted as follows: a municipality with an R_m_ < 1 reflects a lower likelihood of infection to other municipalities, whereas an R_m_ > 1 represents increased likelihood of infection to other municipalities. The time-varying R_m_ was plotted over time and a Generalised Additive Model smoothing spline was fitted to the data to determine trends and smooth data noise.

### 4.7. Ethics

Ethical clearance was granted by the Pan American Health Organization Ethics Review Committee (PAHO-ERC; Ref No. 2014-10-0023) and accepted by the Dominican Republic Ministry of Health. De-identified and aggregated data were used throughout the study, and no further ethical clearance was required.

## 5. Results

### 5.1. Geostatistical Mapping

**Figure 2:**
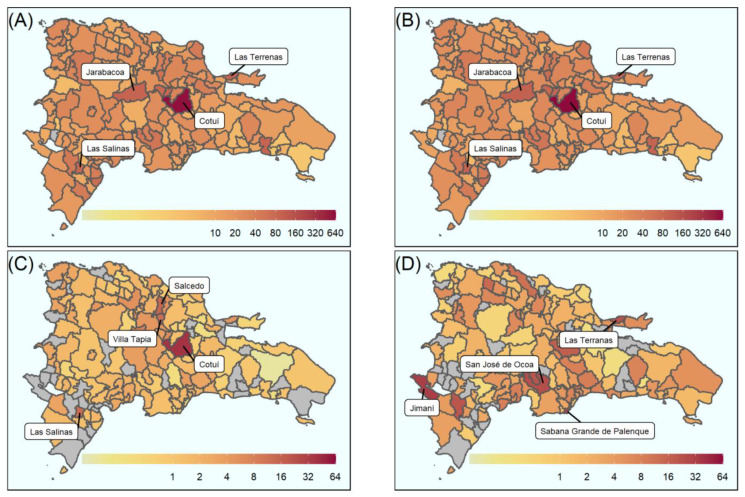
Aggregated spatial distribution of cases over the 2014–2016 epidemic period at the municipal level: (**A**) uncomplicated dengue; (**B**) dengue; (**C**) complicated dengue; (**D**) Zika. Continuous colour scale from white (lowest) through to red (highest) for all images; scales vary. Grey areas indicate no data for those municipalities. All counts per 10,000 population.

Spatial mapping of Zika and dengue was used to determine the highest burden areas for all disease outcomes across the Dominican Republic (Figure 2). Spatio-temporal mapping displays incidence per 10,000 population (non-standardised), while attack rates were standardised according to age and sex with accompanying confidence intervals. These can be seen in Supplementary Table S1 (by province) and Supplementary Table S3 (by municipality), for total dengue, and Supplementary Table S2 (by province) and Supplementary Table S4 (by municipality), for Zika.

The municipality of Cotuí recorded the highest burden for each dengue outcome: 583 (uncomplicated dengue), 39 (complicated dengue) and 622 (total dengue) cases per 10,000 population, respectively (Figure 2), the largest dengue burden of any municipality. Municipalities that also recorded high total dengue burden include Las Terrenas (99 per 10,000 population), Jarabacoa (98 per 10,000 population) and Las Salinas (95 per 10,000 population) (Figure 2B). The burden of severe dengue was also high in Salcedo (15 per 10,000 population), Las Salinas (13 per 10,000 population) and Villa Tapia (12 per 10,000 population) (Figure 2C). The highest burden of Zika incident cases was recorded in the west of the country, Jimaní, with 32 cases per 10,000 population (Figure 2D).

**Figure 3:**
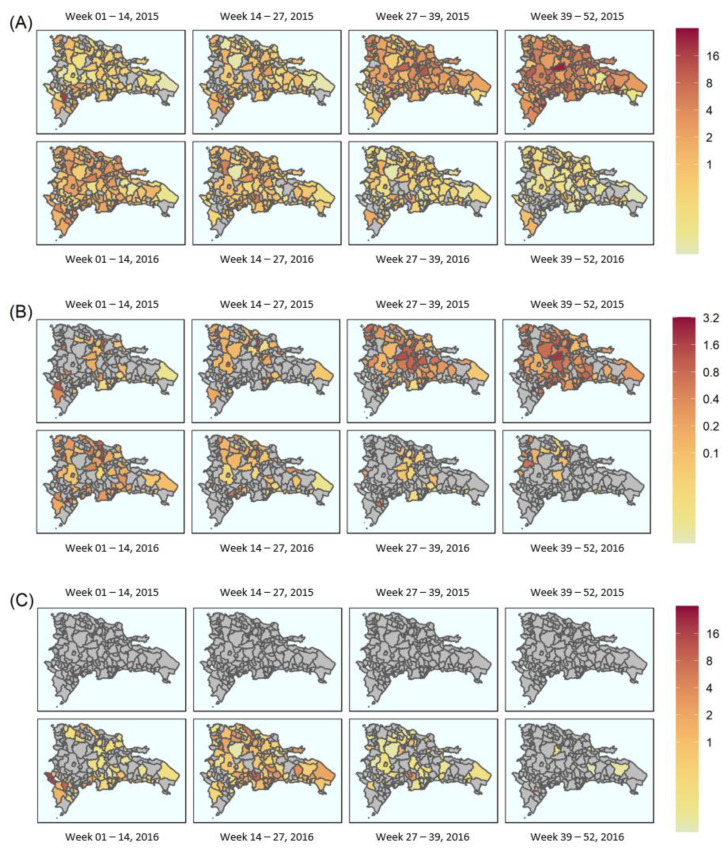
Breakdown of the aggregated spatial distribution of cases over the 2014–2016 epidemic period by epidemiological week at the municipal level: (**A**) dengue cases over 2015 (top row) and 2016 (bottom row); (**B**) complicated dengue cases over 2015 (top row) and 2016 (bottom row); (**C**) Zika cases over 2015 (top row) and 2016 (bottom row). Continuous colour scale from white (lowest) through to red (highest) for all images. Scales vary as shown. Grey areas indicate absence of data. All counts per 10,000 population. Dates are over epidemiological weeks 1–52 for each year, where the year is split into weeks 1–14, 14–27, 27–39 and 39–52.

Three outcomes were also displayed over time and space (Figure 3). Where the outcome was dengue, the highest burden municipalities were Jarabacoa (26 per 10,000 population), Ramón Santana (23 per 10,000 population) and (20 per 10,000 population) (Figure 3A), which occurred between epidemiological weeks 39–52 in 2015. The highest burden of complicated dengue incident cases was recorded in Las Salinas, Villa Tapia and Jarabacoa, with 2 cases per 10,000 population each (Figure 3B) within the same time period.

The first suspected cases of Zika in 2015 were identified during epidemiological weeks 14–27 (2015) and reported in San Cristobal (Figure 3C), while the highest burden of Zika disease was reported in Jimaní (16 cases per 10,000 population: epidemiological weeks 1–15, 2016). In 2016, the peak of Zika cases occurred between epidemiological weeks 1 and 27 (Figure 3C). Throughout weeks 14–27, the greatest burden of Zika disease was recorded in Sabana Grande de Palenque, San José de Ocoa and Sabana Larga (13, 9 and 6 cases per 10,000 population, respectively) (Figure 3C).

### 5.2. Transmission Dynamics

**Figure 4:**
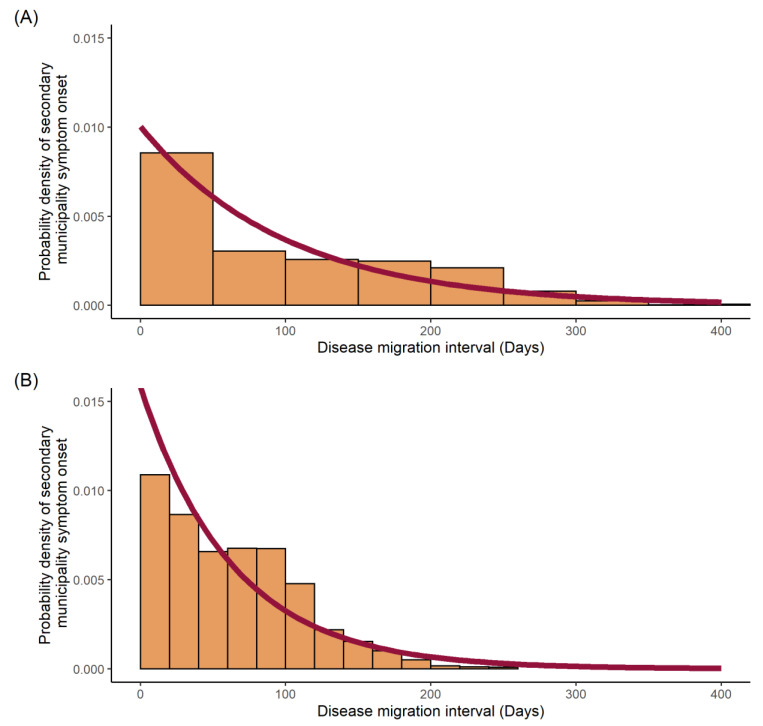
Disease migration interval distribution fitted to an exponential distribution: (**A**) dengue cases; (**B**) Zika cases. Probability density histograms plotted of the disease migration interval represent the distribution of time differences between initial symptom onset within each municipality. Red line represents the maximum likelihood estimation of the exponential distribution describing the data.

The results indicate an exponential distribution between the probability density of secondary municipality symptom onset (infectee) and the time of symptom onset in the infector municipality (Figure 4) for both dengue and Zika. Probability of transmission from infector to infectee municipalities was elevated (~0.1) for both dengue and Zika at the beginning of the epidemic. Probabilities for dengue remained elevated for the first ~50 days before tailing off, whereas probabilities for Zika were high for the first ~125 days before a gradual decline.

The mean disease migration interval, probability of transmission per day (expressed as a rate), standard deviations and log likelihoods for the fitted distributions can be seen in Table 1.

**Figure 5:**
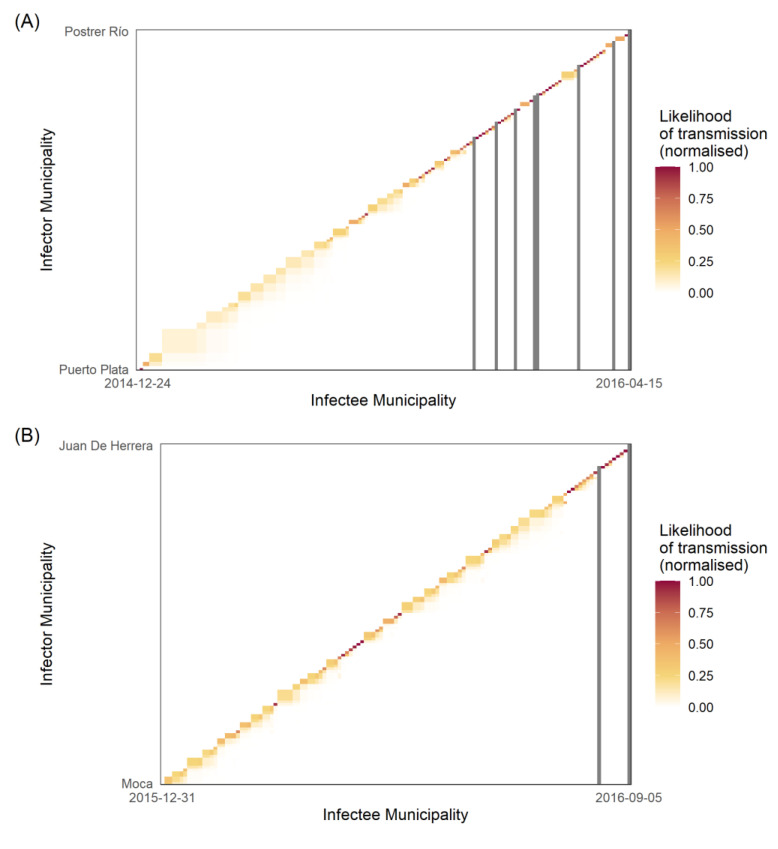
Heatmaps of transmission likelihood of infector–infectee municipal pairs: (**A**) dengue cases; (**B**) Zika cases. The heatmap *X* axis represents all possible infector municipalities ordered by time of initial symptom onset date; the *Y* axis represents all possible infectee municipalities ordered by time of symptom onset. Each square represents the transmission likelihood for said infector–infectee pair. Continuous scale from white (0) to red (1) represents the normalised likelihood of transmission, with white squares indicating no likelihood of transmission. Dark grey lines represent where infectee municipalities were unlikely to be infected by other observed municipalities, and so infection occurred by unobserved disease migration.

**Figure 6:**
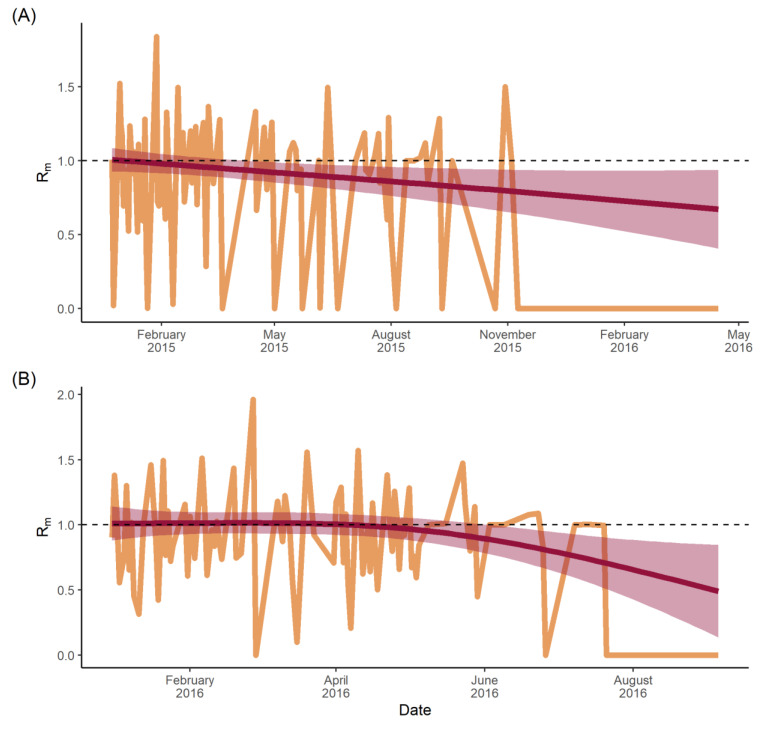
Municipal-specific, time-varying reproduction numbers: (**A**) total dengue cases; (**B**) Zika cases. R_m_ is the number of municipalities a given infector municipality is likely to infect. Red line represents the fitted Generalised Additive Model with smoothing splines and the 95% confidence interval seen as a shaded grey area. Dashed line represents an R_m_ = 1.

The disease migration intervals were used to produce the Wallinga–Teunis matrices (Figure 5) along with the transmission network between infector–infectee municipal pairs for total dengue and Zika (Figure 6).

During the exponential phase of the outbreak, the transmission likelihood matrix for dengue was populated by lower likelihood transmission events compared with the downward curve of the outbreak (i.e., the end of the transmission chain seen in the matrix) (Figure 5A). The matrix also identified seven transmission events with a 0% likelihood of transmission between the infector and infectee, all of which occurred in the latter half of the transmission network. By comparison, the Zika transmission likelihood matrix (Figure 5B) displayed two transmission events with a 0% likelihood of transmission between infector and infectee, both of which also occurred at the end of the transmission network. The transmission chain itself, however, was populated with many high-likelihood transmission events, with few pairwise infector–infectee likelihoods below 50%. In other words, the likelihood of transmission between infector and infectee municipalities increased over time for both dengue and Zika, although the probabilities were higher and earlier for Zika.

To obtain the time-varying R_m_, the sum of the transmission likelihoods for each infectee municipality was calculated and plotted over time, so the R_m_ values were calculated for each transmission event (Figure 6). For total dengue (Figure 6A), there was a linear trend starting at an R_m_ of ~1.0 in January 2015, which decreased to a value of approximately 0.7 by April 2016. Zika (Figure 6B) showed a consistent R_m_ of approximately 1 until just after April 2016 when it began to decline. By September 2016, the R_m_ was ~0.4. Grey areas in Figure 6 represent the 95% confidence intervals, which increase in size over time and correlate with greater uncertainty as the caseload declines.

## 6. Discussion

This research set out to explore the spatio-temporal trends of both dengue and Zika epidemics between 2014 and 2016, and better define disease progression at a municipality level across the Dominican Republic. Retrospective analysis of incident case data was used to map the spatio-temporal distribution of cases. Transmission likelihood matrices for infector–infectee pairs were generated, and the temporal trend in R_m_ was calculated to better understand transmission dynamics over time.

Dengue and Zika attack rates over the entire epidemic period varied substantially across the country, likely a result of known transmission drivers, such as socio-economic conditions and land use [35]. As shown in Figure 3A, the peak of the dengue epidemic occurred during epidemiological weeks 39–52 of 2015, which coincided with the implementation of control efforts, such as fogging and public health campaigns [36], that may have stymied transmission [37]. By contrast, the peak of the Zika epidemic occurred between weeks 14 and 27 of 2016 (Figure 3C). Uncomplicated dengue attack rates were highest in the municipality of Cotuí, at 622 cases per 10,000 population. No other municipality recorded >100 cases per 10,000 population. By contrast, the highest Zika attack rate was recorded in the municipality of Jimaní, at 32 per 10,000 population, and equates to a ~20-fold difference in incidence, demonstrating the serious continuing burden of dengue in the Dominican Republic.

That Jimaní recorded the highest Zika burden in the country is important not only due to the relatively high caseload. Jimaní has a population of 400,000 and shares a land border with Haiti. It has undergone rapid expansion in recent decades and is a hub for the movement of people and goods across the border [38]. Considering the detection of Zika in Haiti as early as 2014 [39], and that Jimaní has become a gateway for larger campaigns in Haiti [40], it is plausible that a number of Zika importation and re-importation events occurred across both sides of the border. This narrative is hypothetically confirmed by spatio-temporal mapping of Zika, showing that the western-most municipalities were affected greatly in the early phases of the epidemic, while the central and eastern regions were affected later. In light of this, increased disease surveillance capacity in Jimaní could offer valuable early warning for disease events across both sides of the border.

### 6.1. Human Mobility and Infrastructure

Human movement between neighbourhoods and commuter cities is known to intensify dengue transmission [41,42]. Indeed, those provinces (Hermanas Mirabal, Sánchez Ramirez and La Vega) that share these characteristics reported relatively high uncomplicated dengue attack rates of 58, 48 and 38 cases per 10,000 population, respectively (standardised for age and sex) (Supplementary Table S1). For Hermanas Mirabal and La Vega, high standardised attack rates correlate with their geographical location. They are connected by primary roads DR-132 and DR-1 to San Francisco de Macoris and Santiago De Los Caballeros, respectively, two of the ten largest cities by population in the Dominican Republic [24,43]. The Sánchez Ramirez province, which includes the Cotuí municipality, is also placed on one of the Dominican Republic’s primary roads (DR-17) between Santo Domingo and San Francisco de Macoris [43]. Accordingly, municipalities and towns along major commuter belts would likely benefit from greater surveillance and public health capacity.

### 6.2. Transmission Chains

The disease migration interval, seen in Figure 4, describes the likelihood of secondary (municipality) infection as a function of the distribution of disease migration intervals for dengue and Zika. This reflects both the infectious period and human mobility. For dengue, the migration interval was heavily skewed towards the first 50 days after symptom onset, in contrast to Zika, which showed a broader distribution over the first 125 days. Given that both pathogens are transmitted via the same *Aedes* vectors, this suggests a more significant role for sexually transmitted Zika [44], at least in terms of transmission drivers throughout the first half of the epidemic.

Wallinga and Teunis [15] first proposed transmission likelihood matrices to identify breaks in transmission chains. In real terms, this equates to the importation of cases, better known as importation events [17]. The international and intra-national movement of people, and the influence of asymptomatic or unreported individuals, can be captured using this methodology, which can help identify both the index case and the source of importations/reintroductions [17]. Using absolute dengue cases, this study identified seven events that had 0% likelihood of transmission between infector and infectee municipalities; in other words, seven importation events (Figure 5A). These occurred during the latter half of the outbreak and constituted a greater number of importation events than the two events observed for Zika. While it is not possible to tease out the origin of each event, the relative frequency of importation events between diseases is not unexpected, given the assumed largely Zika seronegative international population (due to the novel nature of the virus) vs. the global endemicity of dengue [4]. However, it is also possible that these events are related to intra-national introductions, where individuals become infected through inter-municipal contacts, reinforcing the importance of human mobility as a transmission driver, but also potentially through asymptomatic or unreported infections. The implicit assumption here is that a lower R_m_ requires a greater number of importation events to sustain transmission, and vice versa.

The likelihood of transmission increased with time from symptom onset for both Zika and dengue. Heatmaps for dengue (Figure 5A) showed increasing likelihood of transmission between infector–infectee pairs, as a function of symptom onset over time, likely indicating multiple smaller importation events in pockets of less-connected municipalities in rural areas. By contrast, Zika heatmaps demonstrated a more consistent chain of transmission, most likely reflecting a continuous supply of susceptibles infected by two modes of transmission. This is not atypical for Zika, and has been observed in Rio de Janeiro, Brazil, where multiple introductions over a short space of time led to a national crisis [45], since corroborated by phylogenetic analysis linking the strain to French Polynesia [46].

### 6.3. Municipal Reproduction Number (R_m_)

Defining transmission chains and generating time-varying reproduction numbers can provide epidemiologists with valuable information that inform surveillance, control and response. Methodologies used to generate these metrics are established [47,48] and have been used to determine the impact of cattle culls on the transmission dynamics of foot-and-mouth disease in the UK [47]. However, only a small proportion of such probabilistic studies have focussed on arboviruses, with Salje et al., 2016, looking specifically at the transmission dynamics of chikungunya [49]. Independent cascade models [34] have also been used to determine interactions across networks for infectious disease outbreaks, yet these focussed on individuals’ data [17] or were used in the context of social network modelling [50]. Where this study expands the field is in the use of widely available data at a standard geospatial unit—the municipality—to understand the transmission dynamics of infectious diseases, using a newly defined variant of the basic reproduction number: R_m_. 

In this study, dengue R_m_ was recorded as ~1.0 at the start of the epidemic, but immediately and steadily declined throughout (Figure 6A), likely reflecting two factors: (1) that there was a relatively small pool of susceptibles among a highly mobile population in the early phases of the outbreak; and (2) that this pool depleted fairly rapidly as transmission spread from major urban areas before fading throughout less-mobile populations. By contrast, the Zika R_m_ (Figure 6B) remained constant at a value of 1.0 for four months between January and April, pointing to a large pool of susceptibles [51] that were infected steadily as the infection spread throughout the population. Then in May, the R_m_ steadily declined below 1.0, suggesting both a declining pool of susceptibles and a lower force of infection, perhaps as the virus reached less-connected rural areas. This transmission pattern has been observed previously in French Polynesia and the Federated States of Micronesia, where high seroprevalence of IgM antibodies in the local population suggested an acute outbreak that infected three quarters of the population over a similar time scale: four months, from April to July 2007 [52], before tailing off.

The dengue R_m_ observed in this study provides evidence that vector-borne disease spread between administrative locations, and so between populations, can still occur when the effective municipal reproduction number is below 1. While not unusual for endemic diseases, it is surprising that, despite a huge pool of susceptibles and two modes of transmission, Zika transmission never exceeded an R_m_ of 1.5. There may be a number of factors affecting this finding, such as underreporting of infections and geographic misreporting of infections. Nevertheless, the relatively low R_m_ suggests that public health authorities may have more time to respond to novel vector-borne disease outbreaks than initially anticipated. Moreover, the inherent value of R_m_ as a tool to identify high-risk municipalities, as opposed to high-risk neighbourhoods, has practical value given that much of disease reporting is often captured at this larger spatial unit. 

The R_m_, as a novel metric, clearly has a benefit; it operates at a scale that broadly aligns with existing geospatial data collection, thus addressing the fundamental issues surrounding data and spatial heterogeneity described elsewhere [30,53,54]. Operationally, the R_m_ can be used to identify high-burden and high-risk municipalities that necessitate intervention, thereby aligning with early warning and response systems that operate on similar spatial scales [30,55]. However, it should be cautioned that the R_m_ should be used as a floating metric to guide intervention, rather than a binary threshold used to trigger intervention.

### 6.4. Limitations

Estimation of the time-varying R_m_ across the Dominican Republic required the heuristic determination of an optimal distribution to describe the disease migration interval distribution. We used the maximum likelihood estimation of an exponential distribution fit to the data, as seen in Figure 4, which required the assumptions that the data were identical, independent and discrete, and fitted the interval probability density distribution well. This was supported by the log likelihood for each of these models, which were significantly negative, as shown in Table 1, implying a good fit to the data. However, this and the standard deviation for Zika were lower than in dengue, implying the model fitted the dengue epidemic data better than for Zika. This could have been a product of more uncertainty in the exact distribution for Zika due to the smaller sample size of disease migration intervals.

As suspected cases were used in all analyses, there was the potential for misclassification between not only dengue and Zika, but also within the clinical spectrum of dengue, as well as misreporting. All rates were calculated using 2016 census data, so there will be small discrepancies in precision when standardising earlier datasets. Data paucity was an issue for those over 80 years of age, resulting in increased noise and less reliability across results within this age group.

Differences in surveillance intensity and reporting across municipalities may have biased the R_m_; for example, due to weaker surveillance in one municipality vs. another, and where individuals worked and resided in different municipalities. In these scenarios, the R_m_ would have been reduced; however, the impact on these results is reasonably assumed to be small given nationally standardised case definitions and surveillance programmes, and the small proportion of populations who commute large daily distances.

Wallinga–Teunis matrices rely on the temporal product of the disease migration interval distribution, so for these analyses, the distribution was reasonably assumed to be exponential. Furthermore, the matrices themselves are dependent on the completeness of the dataset regarding asymptomatic and unreported infections. Consequently, the clarity of the transmission chain could be honed by incorporating predictions on rates of asymptomatic or unreported cases, and ensuring reporting harmony across geographic sub-units to reduce the biases often present in real-time datasets.

The production of time-varying reproduction numbers assumed complete susceptibility to the viruses within the population, which was valid for Zika, but less so for dengue due to longstanding endemicity. 

## 7. Conclusions

The results of this study characterised the Zika and dengue burden at the municipality level in the Dominican Republic across 2014–2016. Concentrated disease burden within specific municipalities is hypothesised as likely due to the presence of significant transport arteries, both within the Dominican Republic and across the border to Haiti, as a conduit for increased human movement and disease dispersal. Therefore, increased surveillance and targeted public health measures in these municipalities is warranted.

Furthermore, this research highlights the inception of a novel metric used to quantify and determine transmission chains at the municipal level, which can be used to characterise municipality risk, in terms of secondary transmission to neighbouring municipalities. This approach can be generalised to countries worldwide, for multiple infectious diseases, to refine public health responses by targeting municipalities that are significant contributors to disease spread. Finally, this study further reinforces the importance of importation events that drive transmission where R_m_ is below one, and conversely the significance of immune-naïve populations in facilitating disease spread, which require fewer importation events to sustain transmission. Future research should be focused on the refinement of these novel metrics, and then the application of these to characterise municipalities based on a risk system, reflecting variation in R_m_ outputs.

## Figures and Tables

**Table 1:** Fitted disease migration interval distribution parameters.

Parameter	Disease	
Mean disease migration interval (days)	Total dengue	Zika
	99.55	63.28
Rate (probability of transmission/day)	0.01004	0.01580
Standard deviation	0.00009095	0.0001793
Log likelihood	−66,950	−39,670

## Data Availability

All data files are available from the Open Science Framework database (https://osf.io/VYN2B/, accessed on 5 December 2021).

## Supplementary Materials

The following are available online at https://www.mdpi.com/article/10.3390/v14010162/s1, Table S1. Age and sex standardised attack rates of dengue by province. Respective lower and upper 95% confidence intervals are shown. Table S2. Age and sex standardised attack rates of Zika by province. Respective lower and upper 95% confidence intervals are shown. Table S3. Age and sex standardised attack rates of total dengue by municipality. Respective lower and upper 95% confidence intervals are shown. Table S4. Age and sex standardised attack rates of Zika per municipality. Respective lower and upper 95% confidence intervals are shown.
