# Supplementary material for: Novel Epidemic Metrics to Communicate Outbreak Risk at the Municipality Level: Dengue and Zika in the Dominican Republic
Source: Viruses. 2022 Jan 17;14(1):162. doi: 10.3390/v14010162 (PMC8781936; doi:10.3390/v14010162)
Supplement: Supplementary file 1 [file viruses-14-00162-s001.zip › viruses-1520454-supplementary.pdf]

Supplementary Material

Table S1. Age and sex standardised attack rates of dengue by province. Respective lower and upper 95% confidence intervals are shown.

Province of Residence	Standardised Attack Rate	Lower 95% Confidence Interval	Upper 95% Confidence Interval
Hermanas Mirabal	58.07921	57.85644	58.30198
San José de Ocoa	49.20185	48.846	49.5577
Sánchez Ramírez	48.49327	48.36307	48.62347
La Vega	38.80868	38.75974	38.85763
Santiago Rodríguez	38.07062	37.71529	38.42594
Barahona	33.09816	33.00535	33.19097
Duarte	31.81822	31.75006	31.88637
Distrito Nacional	29.4844	29.4635	29.5053
María Trinidad Sánchez	29.00574	28.86502	29.14646
Hato Mayor	25.61707	25.3909	25.84324
Monseñor Nouel	25.23547	25.12044	25.35049
Azua	24.86613	24.7818	24.95045
San Juan	23.91775	23.83329	24.00222
Santiago	23.75802	23.73761	23.77842
Santo Domingo	23.2899	23.28279	23.29702
Monte Cristi	22.7714	22.59429	22.94851
Puerto Plata	20.60499	20.5439	20.66609
Pedernales	19.88183	19.3406	20.42306
Dajabón	19.72239	19.43175	20.01304
Espaillat	18.54744	18.4613	18.63359
Valverde	18.18141	18.06645	18.29636
Peravia	18.0829	17.9858	18.18001
Samaná	17.80326	17.62246	17.98406
San Cristóbal	16.987	16.95681	17.01718
Baoruco	14.92881	14.7493	15.10832
El Seibo	14.84969	14.64179	15.05759
Monte Plata	14.07224	13.9758	14.16868
San Pedro de Macorís	12.76493	12.70028	12.82958
Elías Piña	11.80936	11.54331	12.07541
Independencia	11.66837	11.36343	11.9733
La Altagracia	11.36612	11.30525	11.42699
La Romana	4.964588	4.891426	5.03775

Table S2. Age and sex standardised attack rates of Zika by province. Respective lower and upper 95% confidence intervals are shown.

Province of Residence	Standardised Attack Rate	Lower 95% Confidence Interval	Upper 95% Confidence Interval
San José de Ocoa	21.160	20.773	21.548
Independencia	13.370	13.015	13.724
Distrito Nacional	9.334	9.315	9.353

Santo Domingo	7.291	7.284	7.298
Valverde	7.288	7.171	7.405
Puerto Plata	6.348	6.288	6.408
Azua	5.855	5.756	5.954
San Cristóbal	5.681	5.649	5.714
Hato Mayor	5.430	5.193	5.666
Espaillat	5.358	5.273	5.444
Samaná	5.032	4.846	5.218
La Altagracia	5.003	4.940	5.067
Santiago Rodríguez	4.326	3.957	4.694
Dajabón	3.435	3.115	3.756
Monte Plata	3.138	3.026	3.250
Peravia	3.034	2.932	3.137
Pedernales	3.000	2.347	3.653
Santiago	2.684	2.665	2.703
Monseñor Nouel	2.588	2.473	2.704
La Romana	2.465	2.390	2.541
Hermanas Mirabal	2.254	2.033	2.475
La Vega	1.920	1.872	1.969
Elías Piña	1.770	1.337	2.204
Sánchez Ramírez	1.766	1.628	1.905
El Seibo	1.680	1.445	1.915
Duarte	1.622	1.555	1.690
Barahona	1.620	1.507	1.734
San Juan	1.531	1.428	1.635
San Pedro de Macorís	1.388	1.324	1.453
Monte Cristi	1.334	1.159	1.508
María Trinidad Sánchez	1.223	1.082	1.364
Baoruco	1.116	0.897	1.334

Table S3. Age and sex standardised attack rates of total dengue by municipality.

Respective lower and upper 95% confidence intervals are shown.

Municipality of Residence	Standardised Attack Rate (per 10,000 population)	Lower 95% confidence interval	Higher 95% confidence interval
Jarabacoa	99.80548	99.46349	100.1475
Ramón Santana	89.70886	87.66433	91.75339
Fantino	89.63507	88.68542	90.58471
Jima Abajo	85.40193	84.76547	86.03838
Las Salinas	84.32807	80.65512	88.00103
Villa Tapia	76.6103	75.76196	77.45864
La Ciénaga	61.26256	59.35661	63.16851
San José De Ocoa	56.34095	55.80489	56.87701
Salcedo	55.89944	55.37521	56.42366
Cotuí	52.90987	52.64865	53.17108
Villa González	50.6077	50.0964	51.11899
Cabrera	49.68283	48.84336	50.5223

San Ignacio De Sabaneta	47.72824	47.11682	48.33966
Tenares	46.91322	46.17762	47.64882
Sabana Larga	44.4343	42.25702	46.61158
Mella	43.95176	39.41779	48.48573
Villa Rivas	42.39822	41.82511	42.97133
Enriquillo	40.41046	39.09039	41.73054
Barahona	39.92808	39.71827	40.13789
Cayetano Germosen	36.2763	33.31374	39.23887
Castillo	34.20579	32.9165	35.49509
La Mata	33.82985	33.32753	34.33217
Villa Los Almácigos	33.65289	31.96162	35.34416
Monte Cristi	33.55048	32.73864	34.36232
Tabara Arriba	33.22181	32.13656	34.30706
San Francisco De Macorís	33.00379	32.89722	33.11036
Fundación	32.87632	30.57498	35.17766
Arenoso	31.6767	30.29701	33.0564
Las Matas De Farfán	31.04217	30.60128	31.48306
Hato Mayor del Rey	30.47963	30.16355	30.79572
Rancho Arriba	29.52567	27.21086	31.84048
Bonao	28.53502	28.3801	28.68995
Azua	28.52515	28.32759	28.7227
Nagua	28.4949	28.23753	28.75227
Juan De Herrera	28.18342	26.64899	29.71785
Santo Domingo Norte	27.69646	27.66495	27.72797
Bohechío	26.75297	24.73621	28.76974
Santo Domingo Oeste	26.73674	26.68871	26.78477
Licey Al Medio	26.71107	25.89305	27.5291
La Vega	26.66034	26.58019	26.74048
Puerto Plata	26.08195	25.95665	26.20724
Villa Vázquez	25.68708	24.28856	27.0856
Las Guaranas	25.51559	24.12641	26.90478
Santiago	25.51513	25.48629	25.54397
Sabana Yegua	25.48877	24.52804	26.4495
Pueblo Viejo	25.37458	23.77025	26.97891
Sosua	24.61137	24.21918	25.00355
Vicente Noble	24.41566	23.59058	25.24075
Santo Domingo Este	24.37927	24.3605	24.39804
Guayubín	24.11644	23.56681	24.66607
San Juan	24.09508	23.94515	24.245
El Pino	24.01008	20.64867	27.37149
Samaná	23.23657	22.92029	23.55285
Loma De Cabrera	23.13504	21.94176	24.32832
Yamasá	22.79716	22.48249	23.11182
Mao	22.63276	22.37781	22.8877
Piedra Blanca	22.60557	21.71943	23.4917
San Cristóbal	22.14353	22.06685	22.22022
Pepillo Salcedo (Manzanillo)	22.08113	20.02022	24.14204
Pedernales	21.8935	21.17831	22.60869
Peralta	21.65648	20.44372	22.86924
Laguna Salada	21.46498	20.67118	22.25878

Dajabón	21.4077	20.74168	22.07371
Padre Las Casas	21.26606	20.37922	22.1529
Cambita Garabitos	21.24353	20.68836	21.79869
Las Yayas de Viajama	21.15576	20.11913	22.1924
Moca	20.47941	20.36602	20.59279
Bisonó	20.39457	19.94033	20.84881
Los Alcarrizos	20.32296	20.26306	20.38286
Jaquimeyes	20.25948	15.84741	24.67154
Bajos De Haina	20.13347	19.99304	20.2739
Duvergé	19.97502	18.46921	21.48083
Baní	19.94201	19.82603	20.05799
Río San Juan	19.8371	18.44851	21.2257
Los Hidalgos	19.81484	18.04952	21.58016
El Peñón	18.89265	14.26395	23.52135
Consuelo	18.70276	18.0918	19.31372
Cabral	18.43594	17.23146	19.64042
El Factor	17.73807	16.92954	18.54659
Tamboril	17.57079	17.19646	17.94513
Neiba	17.0401	16.54895	17.53126
Sabana De La Mar	16.99978	15.80979	18.18976
Monción	16.88539	15.04676	18.72402
Partido	16.86654	13.86123	19.87185
Pedro Brand	16.85734	16.64279	17.07189
Miches	16.80266	15.95822	17.6471
Villa Jaragua	16.42362	14.81411	18.03314
Tamayo	15.92995	15.27947	16.58042
Villa Montellano	15.47258	14.49432	16.45085
Sabana Grande De Boya	15.02801	14.45046	15.60555
Las Charcas	14.93584	13.30949	16.56218
Pimentel	14.58927	13.48946	15.68907
Santa Cruz de El Seibo	14.26439	13.98758	14.54121
Oviedo	14.10137	11.79814	16.40459
Yaguate	14.01328	13.60936	14.41719
Villa Isabela	13.76339	12.5372	14.98958
Bánica	13.58093	10.6233	16.53855
Comendador	13.54216	12.95233	14.132
Estebanía	13.51789	10.20601	16.82978
Los Ríos	13.45697	11.05918	15.85476
San Antonio De Guerra	13.36972	12.99537	13.74407
Hondo Valle	12.9614	11.26777	14.65502
El Cercado	12.85318	11.92014	13.78623
Castañuelas	12.64453	11.40537	13.8837
Esperanza	12.28771	11.99754	12.57787
Higüey	12.26903	12.20223	12.33583
San José De Las Matas	12.16991	11.65137	12.68846
Cevicos	11.80692	10.36058	13.25327
Guananico	11.76473	7.921584	15.60787
Las Terrenas	11.5613	10.5313	12.59131
Polo	11.50662	8.687499	14.32574
El Llano	10.96535	9.174346	12.75636

Constanza	10.89276	10.57411	11.21142
Sánchez	10.68355	9.908002	11.45909
Gaspar Hernández	10.62947	10.10862	11.15031
San Pedro De Macorís	10.51065	10.41208	10.60922
Vallejuelo	10.47525	8.895904	12.05459
Monte Plata	10.4132	10.00501	10.8214
Santo Domingo de Guzmán	10.2152	10.20796	10.22244
Maimón	9.692846	8.692952	10.69274
Boca Chica	9.677225	9.563648	9.790802
Nizao	9.432479	8.772205	10.09275
Janico	9.330062	8.110934	10.54919
Imbert	9.234064	8.329125	10.139
Galván	9.135609	8.016497	10.25472
Paraíso	9.129083	8.076553	10.18161
Puñal	8.974125	8.534393	9.413857
Jimaní	8.950573	8.027251	9.873896
Altamira	8.907666	7.743731	10.0716
Villa Altagracia	8.794135	8.593711	8.99456
Luperón	8.506038	7.315192	9.696883
Bayaguana	8.400559	7.81252	8.988598
Juan Santiago	8.099237	0.161985	16.03649
Guayabal	7.956494	4.057812	11.85518
La Descubierta	7.743339	5.575204	9.911474
Restauración	7.602448	5.473763	9.731134
Las Matas De Santa Cruz	7.171422	5.414424	8.928421
La Romana	7.07478	6.940153	7.209407
El Valle	7.068844	4.297857	9.839831
Sabana Iglesia	6.993931	5.470808	8.517054
Peralvillo	6.794156	5.906386	7.681926
Eugenio Maria De Hostos	6.708117	3.42114	9.995094
Guayacanes	6.471506	5.203091	7.739922
Pedro Santana	5.994467	3.057178	8.931756
Los Cacaos	5.894755	4.244224	7.545287
Quisqueya	5.81898	4.868546	6.769413
Jamao Al Norte	5.21818	2.661272	7.775088
Los Llanos	4.373047	3.51593	5.230164
San Gregorio De Nigua	4.073791	3.503461	4.644122
Sabana Grande De Palenque	3.363778	2.264944	4.462612
Cristóbal	3.129884	1.085027	5.174742
Guaymate	2.681658	1.630448	3.732868
Villa Hermosa	2.480608	2.286128	2.675088
San Rafael Del Yuma	2.220714	1.495281	2.946148
Postrer Río	2.05144	-1.96938	6.072261

Table S4. Age and sex standardised attack rates of Zika per municipality. Respective lower and upper 95% confidence intervals are shown.

Municipality of Residence	Standardised Attack Rate (per 10,000 population)	Lower 95% confidence interval	Higher 95% confidence interval
Jimani	32.3110079	31.199962764	33.4220531
Sabana Grande De Palen que	31.1635729	29.988945902	32.3381999
San Jose De Ocoa	25.6569420	25.085491882	26.2283920
Sabana Larga	24.9988175	22.419992094	27.5776429
Mella	22.1680593	16.736884748	27.5992338
Laguna Salada	13.8565123	13.057725133	14.6552995
Los Alcarrizos	11.9794935	11.915862626	12.0431245
Puerto Plata	11.4667693	11.347854645	11.5856839
Azua	8.8848804	8.658719782	9.1110410
Yamasa	8.5608894	8.179540673	8.9422381
Santo Domingo Norte	8.4313207	8.399107466	8.4635339
Sabana Iglesia	8.3813333	7.012382194	9.7502844
Villa Los Almacigos	7.8986302	5.963465804	9.8337946
Hato Mayor del Rey	7.5330013	7.204897261	7.8611054
San Cristobal	7.3748640	7.296305708	7.4534224
Santo Domingo Este	6.9967543	6.979329043	7.0141795
Moca	6.6630892	6.552414200	6.7737643
Mao	6.5777972	6.334542791	6.8210516
Maimon	6.4023271	5.356613639	7.4480405
Estebania	6.1978345	2.148582610	10.2470863
Boca Chica	6.1964392	6.073762201	6.3191162
Pueblo Viejo	5.9857849	4.030428520	7.9411413
Guaymate	5.7685724	4.355272143	7.1818726
Samana	5.6791387	5.361106943	5.9971705
Janico	5.6002079	4.602352682	6.5980631
Esperanza	5.5881001	5.275166493	5.9010337
Dajabon	5.5087043	4.833888002	6.1835205
Las Yayas de Viajama	5.4675100	4.127970032	6.8070499
Sanchez	5.2444350	4.453735608	6.0351345
Higuey	5.2339712	5.165580662	5.3023618
Santo Domingo Oeste	4.9618576	4.916623941	5.0070913
Cambita Garabitos	4.8807317	4.242982784	5.5184807
San Ignacio De Sabaneta	4.7929775	4.166695081	5.4192599
Las Salinas	4.7902755	0.095805510	9.4847455
Sabana Yegua	4.7736030	3.604070248	5.9431357
Luperon	4.5556290	3.280052865	5.8312051
El Pino	4.4579821	1.545433789	7.3705304
Ramon Santana	4.3993225	2.243654453	6.5549905
La Descubierta	4.1547668	1.440319165	6.8692145
Bajos De Haina	4.0324240	3.886061953	4.1787861
Tabara Arriba	4.0215527	2.707845475	5.3352599
Las Guaranas	4.0192872	2.706320080	5.3322544

Duverge	3.9938524	2.428262244	5.5594425
El Llano	3.9517350	0.079034700	7.8244353
Pedro Brand	3.9407016	3.706647803	4.1747554
Pedernales	3.8533242	3.014155784	4.6924925
Santo Domingo de Guzm an	3.5813698	3.574236191	3.5885034
Bani	3.3054351	3.185460033	3.4254101
Villa Tapia	3.0550819	2.199658977	3.9105048
Yaguate	2.9979435	2.545945881	3.4499412
Las Terrenas	2.8254353	1.717864634	3.9330059
Santiago	2.8209334	2.794975507	2.8468913
Cayetano Germosen	2.8138487	0.056276973	5.5714204
Neiba	2.7419134	2.144785610	3.3390412
Barahona	2.7377511	2.493842378	2.9816598
Salcedo	2.6390676	2.121810327	3.1563248
La Vega	2.6375850	2.559256741	2.7159133
Comendador	2.6288381	1.598333586	3.6593427
Peralta	2.5874098	1.319578974	3.8552405
Guayubin	2.5377422	1.985078332	3.0904060
Fantino	2.4828577	1.509577508	3.4561380
La Romana	2.4590175	2.332183957	2.5858510
Guayacanes	2.4273391	1.237942933	3.6167352
Licey Al Medio	2.4193452	1.629025784	3.2096647
Villa Altagracia	2.3683721	2.136271672	2.6004726
Bonao	2.2882133	2.133561601	2.4428649
Villa Gonzalez	2.2798786	1.783371703	2.7763855
San Juan	2.2505690	2.074124375	2.4270136
La Mata	2.2182605	1.674786661	2.7617343
Bisono	2.1160769	1.655242388	2.5769114
San Francisco De Macoris	1.9711141	1.869446083	2.0727821
Castanuelas	1.9551102	0.677771522	3.2324488
Villa Hermosa	1.9428478	1.731293231	2.1544023
Tamboril	1.9257006	1.548263248	2.3031379
Guayabal	1.9073927	-1.831097011	5.6458825
Peralvillo	1.8898764	0.963836984	2.8159159
Santa Cruz de El Seibo	1.8710630	1.565456042	2.1766700
San Pedro De Macoris	1.5842176	1.490124677	1.6783105
Nagua	1.5703194	1.313833891	1.8268049
Altamira	1.5438997	0.030877994	3.0569214
Pedro Santana	1.5394848	-1.477905401	4.5568750
Cotui	1.5062534	1.237866434	1.7746404
San Antonio De Guerra	1.4871201	1.070726469	1.9035137
Nizao	1.4756720	0.752592713	2.1987513
Castillo	1.4643032	0.029286064	2.8993203
Fundacion	1.4457480	-1.387918078	4.2794141
San Rafael Del Yuma	1.3746690	0.701081169	2.0482567
Piedra Blanca	1.3607211	0.471716637	2.2497255
El Factor	1.3308969	0.461377576	2.2004161
Punal	1.2720042	0.856482823	1.6875256
Jima Abajo	1.2345934	0.629642633	1.8395442
Tenares	1.1261471	0.390397665	1.8618966

Miches	1.1019171	0.022038343	2.1817959
Monte Plata	1.0452904	0.635536553	1.4550442
Imbert	1.0206539	0.020413078	2.0208947
Los Cacaos	1.0094193	-0.969042510	2.9878811
Vicente Noble	1.0040338	0.020080675	1.9879869
Cristobal	1.0002930	-0.960281281	2.9608673
Padre Las Casas	0.9838419	0.019676838	1.9480069
Las Charcas	0.9819012	-0.942625159	2.9064276
Restauracion	0.9803555	-0.941141299	2.9018523
San Gregorio De Nigua	0.9420560	0.326579411	1.5575326
Enriquillo	0.9369993	-0.899519289	2.7735178
Sosua	0.9251375	0.562483600	1.2877914
Cevicos	0.9205966	-0.883772781	2.7249661
Las Matas De Farfan	0.8601461	0.298183977	1.4221082
Gaspar Hernandez	0.8527920	0.295634556	1.4099494
Las Matas De Santa Cruz	0.8518128	-0.817740285	2.5213659
Loma De Cabrera	0.8265447	-0.793482887	2.4465722
Juan De Herrera	0.8032091	-0.771080766	2.3774990
Los Hidalgos	0.7504047	-0.720388514	2.2211979
Constanza	0.6942031	0.354043562	1.0343626
Villa Isabela	0.6891028	-0.661538716	2.0397444
Rio San Juan	0.6527960	-0.626684125	1.9322761
Villa Vazquez	0.6285566	-0.603414334	1.8605275
Cabrera	0.5035959	-0.483452092	1.4906440
Monte Cristi	0.4637674	-0.445216695	1.3727515
San Jose De Las Matas	0.4387227	0.008774453	0.8686709
Jarabacoa	0.4355955	0.151006446	0.7201846
Tamayo	0.3854663	-0.370047622	1.1409802
Consuelo	0.3807781	-0.365546933	1.1271030
Villa Rivas	0.3642234	-0.349654457	1.0781012
Bayaguana	0.3230117	-0.310091264	0.9561147
Sabana Grande De Boya	0.3042786	-0.292107421	0.9006645
Arenoso	NA	NA	NA
Banica	NA	NA	NA
Bohechio	NA	NA	NA
Cabral	NA	NA	NA
El Cercado	NA	NA	NA
El Penon	NA	NA	NA
El Valle	NA	NA	NA
Eugenio Maria De Hostos	NA	NA	NA
Galvan	NA	NA	NA
Guananico	NA	NA	NA
Haiti	NA	NA	NA
Hondo Valle	NA	NA	NA
Jamao Al Norte	NA	NA	NA
Jaquimeyes	NA	NA	NA
Juan Santiago	NA	NA	NA
La Cienaga	NA	NA	NA
Los Llanos	NA	NA	NA
Los Rios	NA	NA	NA

Moncion	NA	NA	NA
Otro Extranjero	NA	NA	NA
Oviedo	NA	NA	NA
Paraiso	NA	NA	NA
Partido	NA	NA	NA
Pepillo Salcedo (Manzani llo)	NA	NA	NA
Pimentel	NA	NA	NA
Polo	NA	NA	NA
Postrer Rio	NA	NA	NA
Quisqueya	NA	NA	NA
Rancho Arriba	NA	NA	NA
Sabana De La Mar	NA	NA	NA
Vallejuelo	NA	NA	NA
Villa Jaragua	NA	NA	NA
Villa Montellano	NA	NA	NA
